# Real world evidence of the use of metamizole (dipyrone) by the Brazilian population. A retrospective cohort with over 380,000 patients

**DOI:** 10.31744/einstein_journal/2022AO6353

**Published:** 2022-04-25

**Authors:** Henry Sznejder, Caroline Amand, Andrew Stewart, Ricardo Salazar, Wanessa Alessandra Ruiz Scala

**Affiliations:** 1 UnitedHealth Group São Paulo SP Brazil UnitedHealth Group, São Paulo, SP, Brazil.; 2 Sanofi Pharmaceuticals Paris France Sanofi Pharmaceuticals, Paris, France.; 3 Sanofi Pharmaceuticals Panamá Panamá Sanofi Pharmaceuticals, Panamá, Panamá.; 4 Sanofi Pharmaceuticals São Paulo SP Brazil Sanofi Pharmaceuticals, São Paulo, SP, Brazil.

**Keywords:** Dipyrone, Pain, Fever, Antipyretics, Drug combinations, Monotherapy

## Abstract

**Objective:**

To determine under which health conditions metamizole (dipyrone) is used as a single drug or as fixed-dose combination.

**Methods:**

Two retrospective cohorts of Brazilian patients treated with metamizole between January 2015 and December 2017 were analyzed: a metamizole-based cohort (Cohort 1) and a symptoms-based cohort (Cohort 2). Anonymized patient data was obtained from Amil Clinical Data Warehouse. The number of patients with symptoms was described by age and sex.

**Results:**

The sample size of the two cohorts consisted of 384,668 patients. In patients using metamizole (Cohort 1), the most common reason for medication was the treatment of some form of pain (81%), followed by fever (19%). Headache was the most common (19%) specified pain class, followed by sore throat (8%), muscular pain (6%), and abdominal pain (5%). In adult patients (n=276,279; 71.8%), metamizole was used as a monotherapy or associated with another drug, for any sort of pain, in over 88% of the patients. General pain was the main reason for metamizole use in children (61%).

**Conclusion:**

Real world evidence to evaluate Brazilian patients’ therapeutic options is unusual and yet to be more explored using digital tools enabling better data registration. The present study confirmed that metamizole is widely used as a non-anti-inflammatory drug, and also showed the management of pain and fever as the most frequent indications in all age groups studied.

**Registry in Clinical Trials Database:** REBEC Database: 10507

## INTRODUCTION

Metamizole, also known as dipyrone, is a non-narcotic pyrazolone derivative. It is one of the most widely used drugs in Brazil for pain and fever relief. In Brazil, metamizole is prescription free and can be purchased over the counter (OTC) as Novalgina^®^, Dorflex^®^, Lisador^®^, Neosaldina^®^ and Buscopan^®^ composto.^(1)^ Although used by hundreds of millions of patients worldwide, its mechanisms of action are still not completely clear.^(2)^ Metamizole has analgesic, antipyretic, and spasmolytic effects, and presents more favorable gastrointestinal tolerability when compared to other nonsteroidal anti-inflammatory drugs (NSAIDs).

However, controversy exists in the medical literature as for the safety of metamizole. This is reflected in its regulatory clearance and adoption in different countries. Although metamizole is the most popular analgesic agent in some European countries and in Latin America, it has been banned from other markets such as the United States (US) and the United Kingdom (UK) because health authorities judged that the risk of severe adverse events (especially agranulocytosis) outweighs its benefits.^(3)^

A multicentric international case-control study aimed to identify the most common risk factors for agranulocytosis (LATIN Study). The incidence of agranulocytosis was 0.38 cases per one million person-years (0.35 in Brazil). The use of medications associated to agranulocytosis was significantly more frequent in patients with agranulocytosis than controls, mainly antithyroid drugs. However, no significant relation of agranulocytosis and previous exposure to metamizole was found.^(4,5)^

The use of real world data (RWD) has grown tremendously in the last few years and a bright future is expected in the healthcare area. It grows not only supported by the opportunity to capture data with greater accuracy through electronic records and other medical devices, but also the prospect of processing all these data and transforming them in valuable information to better understand the responses and interactions in a determined population. Thus, performing targeted analyses and moving away from perceptions and broad extrapolations to the facts about patient journeys and outcomes.^(6)^

Real world data are also expected to not only impact the way we develop clinical programs and trial design, with hydrides sources of information from clinical research data and real-life inputs, but also promote evaluation of mature drugs in the market and the way people are used to consuming them, gathering data from aspects of daily life. Therefore, RWD has great value to demonstrate ongoing safety and drive decisions to improve clinical development via enabling translational research, better understanding of drug pathways, higher value population and product profiles, as well as trial simulation and recruitment. Hence, real world evidence (RWE) studies may deliver deeper insights about patient care, treatment pathways, and drug effectiveness than previously thought possible. Although highly valued, the major challenges for RWD are the failures to capture biomarker data and consistent disease assessment, with a need for future improvements.^(7)^

## OBJECTIVE

To determine under which health conditions metamizole is used in real life as a single drug or in combination, in addition to comparing metamizole pattern of use for different conditions.

## METHODS

This article was written according to the Strengthening the Reporting of Observational Studies in Epidemiology (STROBE) guide.^(8)^ This is a descriptive, retrospective, observational cohort study using anonymized data from Amil Clinical Data Warehouse (CDW), designed to describe demographic and clinical characteristics of patients in Brazil treated with metamizole, conducted between January 2015 and December 2017 to investigate two study cohorts: a metamizole-based cohort (Cohort 1) and a symptoms-based cohort (Cohort 2).

Patients using Amil’s network have their individual medical files recorded in the existing electronic health record system (EHR), available for Population Health Management (PHM) and research initiatives according to terms and consent of data sharing. All data used in the present study were collected from CDW. The EHR information analyzed consists of a combination of structured and unstructured data, given that part of the record is captured in free-text fields, including most of the prescriptions, symptoms, lab results, and physicians’ notes. To extract the information from the EHR across thousands of records, the application of Natural Language Process (NLP) techniques to the unstructured fields was used to capture relevant clinical information from free-text medical records.

This study was approved by a Research Ethical Committee from *Hospital Pró-Cardíaco*, CAAE: 05738919.6.0000.5533 protocol #3.119.996 and it was given an exemption of the individual Informed Consent Form, since data collection was anonymized and indirectly performed from the medical records. The sample was divided into two cohorts. Cohort 1: metamizole-based cohort - with at least one record of metamizole prescription during the observed period, and Cohort 2: symptoms-based cohort - with at least one record of headache, migraine or fever. Exclusion criteria for both cohorts included patients under oncologic treatment during the period of the study, rheumatic and autoimmune disorders, pregnancy, history of metamizole allergy, including anaphylactic reactions, hematological history and deficiency of glucose-6-phosphate dehydrogenase (G6PD).

Several subgroups were formed from patients identified in Cohort 1: drug used, age group, symptoms. Patient subgroups in Cohort 2 were assigned based on the type of symptoms and type of drug in their medication records.

As this is a retrospective study, we analyzed all patients who met the eligibility criteria and there was no pre-specified sample size or power calculation.

### Cohort 1 analyses

For Cohort 1 (all metamizole users) analyses, descriptive statistic was used to summarize metamizole use by patients within each age category, both overall and for those using monotherapy and fixed-dose combination forms. The number of patients with each symptom was determined, and descriptive statistic was used to calculate the number and percentage of patients that used metamizole, as well as other analgesic and antipyretic drugs, within each symptom. The analysis was repeated for the pediatric subgroup looking for the same symptoms and drugs found in the adult group.

### Cohort 2 analyses

For Cohort 2, patient counts were summarized for the subgroups of patients with fever and those with headaches/migraines. For each drug subgroup (metamizole, ketoprofen, acetaminophen, ibuprofen, and AAS), the number of patients within each symptoms-based subgroup was determined.

## RESULTS

A total of 455,834 patients were identified as having at least one record of use of metamizole during the study period (36 months). The exclusion from this population included 16% with an invalid identification, indication of allergy or those who denied having used metamizole (n=37,795), or at least one exclusion criteria (n=13,112). The final Cohort consisted of 384,668 distinct patients.

### Cohort 1

Table 1 shows a summary of the baseline demographic characteristics of patients included in the metamizole Cohort. The majority were women (59.0%) and most patients were aged <40 years (66.2%). Children represented 28.2%.

**Table 1:** Demographic characteristics of patients in the metamizole-based cohort (Cohort 1)

	Metamizole cohort (Cohort 1) (n=384,668)
Age group (years), n (%)	
0-18	108,389 (28.2)
19-39	146,021 (38.0)
40-59	91,008 (23.7)
60-79	30,583 (7.9)
80+	8,667 (2.2)
Sex, n (%)	
Female	227,080 (59.0)
Male	157,588 (41.0)

Peak of incidence of metamizole use was observed up to fourth decade of life for both men and women.

**Figure 1:**
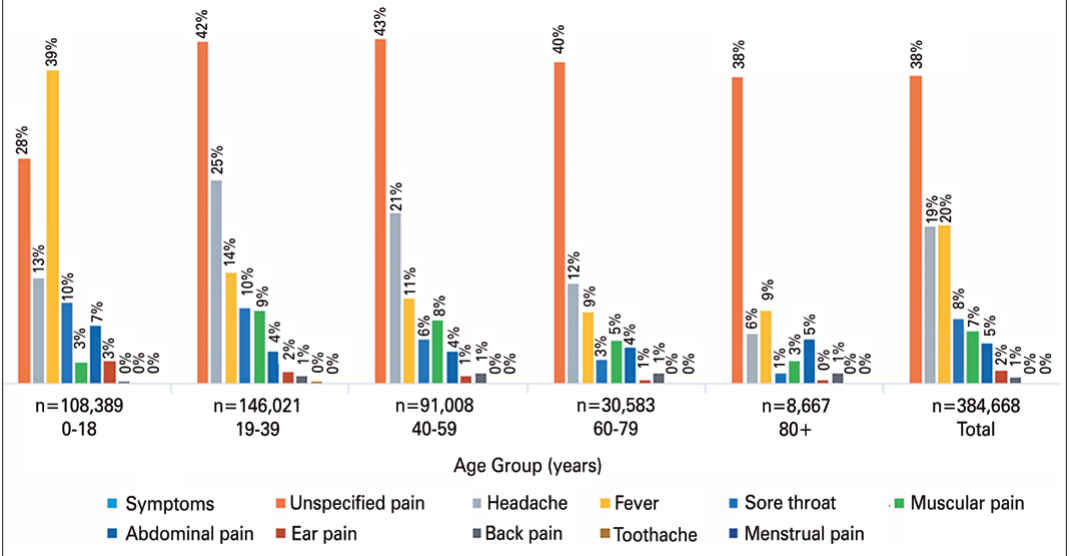
Associated symptoms and conditions with metamizole use by Age Group

In patients who used metamizole (Cohort 1), the most common reason for medication was the treatment of some form of pain (81%), followed by fever (19%). Headache was the most common specified pain class (19%), followed by sore throat (8%), muscular pain (7%), and abdominal pain (5%). As shown in figure 1, evaluating by age groups, adult patients used metamizole more frequently to treat headache, fever and sore throat, followed by myalgia and abdominal pain. The use for fever declined among older adults. The main reason for children to use metamizole was fever (39%) and general pain (28%). The conditions flu/influenza, arbovirus, and/or sinusitis were identified in <3% of the cases. Only 0.9% of the studied population used metamizole for some kind of arbovirus infection (namely dengue or chikungunya), with epidemic proportions in Brazilian urban areas during the study period.

In adult patients (71.8%), metamizole was used as a monotherapy or associated with another drug, for any sort of pain, in over 88% of the patients and for fever in 12% of the cases. While pain remained the main reason for metamizole use in children (61%), it was used for the management of fever in 39% of the cases.

**Figure 2:**
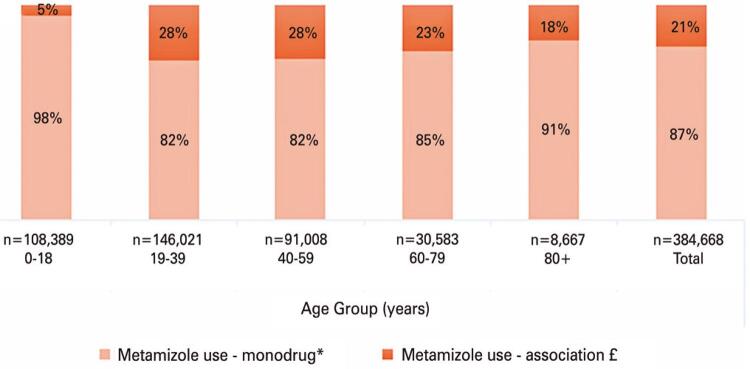
Metamizole use as a monotherapy or in association

As presented in figure 2, metamizole was reported as a monotherapy in most patients of the Cohort (87%), reaching 98% in patient aged <19 years old. Metamizole was used in association with other analgesic and antipyretic drugs in almost one quarter of the patients aged between 19 and 79 years old.

* Monodrug: includes Dipyrone, Novalgina^®^, Anador^®^ £ Fixed-dose combinations includes Neosaldina^®^ (dipyrone, caffeine, isometheptene), Dorflex^®^ (dipyrone, caffeine, orphenadrine), Lisador^®^ (dipyrone, adiphenine, promethazine), Cefaliv^®^ (dipyrone, caffeine, dihydroergotamine), Benegrip^®^ (dipyrone, caffeine, chlorphenamine), Buscopan^®^ composto (dipyrone, scopolamine butylbromide).

Other analgesic and antipyretic drugs associated with metamizole were used by patients, such as ketoprofen (7.5%), acetaminophen (4.2%), and ibuprofen (3.5%). In patients aged 60 or more, ibuprofen was less frequently used (1.8% in patients aged 60-79 and 1.0% in patients aged 80 or more), while aspirin use was increased (4.4% in patients aged 60-79 and 6.5% in patients aged 80 or more).

For the treatment of pain, when used in combination, ketoprofen was the most commonly associated drug with metamizole. For the treatment of fever, when used in combination, acetaminophen was the most frequently associated drug (6.1%) followed by ketoprofen (5.1%).

Metamizole users with headache symptoms (n=6,036) were those identified as having a systematic usage of other analgesic and antipyretic drugs, 43.4% used ibuprofen and 51.6% used ketoprofen.

In children, metamizole was used as a monotherapy in 98% of the cases. Acetaminophen and ibuprofen were the only other drugs associated with metamizole for the treatment of fever or pain.

Table 2 shows the distribution of the following symptoms: generalized pain, pain with fever, pain without fever, fever without pain, as well as other subgroups of interest.

**Table 2:** Distribution of symptoms

Symptoms	Metamizole cohort (Cohort 1) n=384,668 (%)
Generalized pain	146,892 (38.2)
Pain with fever	34,769 (9)
Pain without fever	111,779 (29.1)
Fever without pain	40,695 (10.6)
Headache	74,551 (19.4)
Flu, cold symptoms, sinusitis, and sore throat	1,162 (0.3)
Patients with arbovirus	3,032 (0.9)

In the subgroup focusing on generalized pain (38.2%), ketoprofen (6%) was the most common medication prescribed with metamizole, followed by acetaminophen (5%) and ibuprofen (4%). Prescribed ages vary widely, but 20-49 years are the most common, with an apparent peak in the 30-39 age group. The most common symptoms associated with prescriptions of metamizole were headaches (48.9%), followed by fever (23.8%) and muscular pain (17%).

In patients with pain and without fever (29.1%), Dorflex^®^ prescriptions (15.5%) were almost three-times as common as the next most common drug, ketoprofen (5.8%), and headaches were again the most common symptom associated with metamizole prescriptions.

In patients with symptoms of headache (19.4%), Dorflex^®^ (14.8%) was the most common pain medication prescribed with metamizole, followed by Neosaldina^®^ (9.7%). The most common specific symptoms were fever (23.9%), muscular pain (16.1%), and abdominal pain (4.8%).

In patients with flu, cold symptoms, sinusitis and sore throat (0.3%), acetaminophen (8.4%), ketoprofen (7.4%) and ibuprofen (6.8%) were prescribed at similar frequencies as in the other symptoms-based subgroups; however, diclofenac (2.5%) was considerably more common in this subgroup, associated with metamizole as well.

In patients with evidence of arbovirus infections (0.9%), acetaminophen was the most prescribed drug alongside metamizole. The most common specific symptoms were fever (35.5%), headache (26.2%), and muscular pain (26%).

The analyses described for Cohort 1 showed the relative importance of fever and headache as frequent symptoms associated with the use of metamizole.

### Cohort 2

This symptom-based cohort (Cohort 2) allowed the assessment of medication use in patients with at least one record of headache or fever.

As presented in table 3, among the 19,902 identified patients in Cohort 2, fever was the most frequent symptom (51.7%).

**Table 3:** Patient identification

Total symptomatic patients (n=19,902)		
Fever (%)	Fever + Headache (%)	Headache (%)
10,300 (51.7)	3,324 (16.7)	6,278 (31.5)
All fever group*: 13,624		
	All headache group*: 9,602	

* For these two groups, patients having Fever + Headache have doubled counted for each respective group fever and headache.

As presented in table 4, among the 19,902 identified patients in Cohort 2, approximately 63.6% of the patients had no record of medication used for the mentioned symptoms. This proportion of patients using medicines was similar between symptoms, around 33% of symptomatic patients used at least one drug.

**Table 4:** Use of medication

	Symptoms			

	Fever 10,300 (%)	Fever + Headache 3,324 (%)	Headache 6,278 (%)	Total 19,902 (%)
Drug use	3,459 (33.6)	1,754 (52.8)	2,039 (32.5)	7,252 (36.4)
No drug	6,841 (66.4)	1,570 (47.2)	4,239 (67.5)	12,650 (63.6)

**Figure 3:**
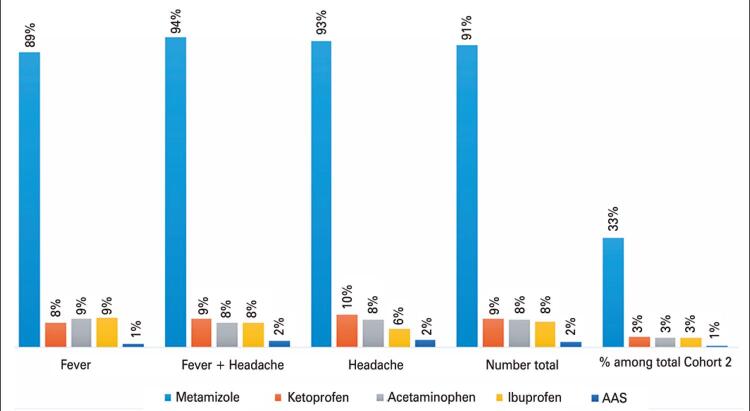
Distribution of patients with fever and/or headache, and the identification of medication associated with symptom (Cohort 2)

In patients with a recorded use of medication (36.4%), metamizole was by far the most common used drug (91.4%), followed by ketoprofen, acetaminophen, and ibuprofen (8.6%, 8.3%, and 7.7%, respectively). The same trend was observed by type of symptom as shown in figure 3.

## DISCUSSION

This real world study comprised two cohorts, one consisting of patients with evidence of metamizole use, and another consisting of patients with symptoms of pain, different types of headache such as migraine, or fever. The high number of patients with records of at least one episode of metamizole use in the described population demonstrate the high utilization rate, good tolerance and widespread use of the drug for the treatment of pain and fever in Brazil. These data also helped to validate a pattern observed in the literature, that for patients with the two most prevalent symptoms (headache and fever), metamizole was prescribed more frequently compared to other available drugs.

A recent Brazilian survey studied the use of analgesics (under prescription and OTC) by a randomly assigned population of more than 41,000 persons in all areas of the country. The authors observed that one in five people had used at least one analgesic drug during the past 15 days, and metamizole was the most common used drug, followed by acetaminophen and diclofenac. The prevalence of analgesic use was higher in women patients, people with higher education levels, with private health insurance, and those aged 60 or over.^(9)^

The fact that this RWE was extracted from a supplementary health coverage may be a source of bias when trying to extrapolate the data to the Brazilian population. However, similar data were found in other studies, which verified that women use analgesics more frequently than men.^(10,11)^ This can be explained by the greater frequency of pain conditions among women, mainly in menstrual period, that strongly influence the use of analgesics.^(10,11)^

One additional source of potential bias (especially in Cohort 2) is the low number of patients that had a record of medication taken for fever or headache episodes (approximately one in three episodes only), with insufficient data on fever management. That can be explained by the fact that most cases in that cohort came from outpatient settings with no urgent care. However, we have no reason to believe the real pattern of medicine choice to be different from that found in the described results.

Attention is always required when interpreting results of comparative observational studies given the lack of randomization and subsequent biases introduced in an observational design. However, some of the findings of the present study were consistent with the Brazilian literature, especially with a study showing that ~20% of patients who used analgesics took at least two associated analgesic drugs.^(9)^ In this cohort, 20.7% of patients who had a record of metamizole use had other associated drug.

In line with Brazilian data, the present study (especially Cohort 2) showed a higher prevalence of metamizole than NSAIDs and was much higher than the use of opioids. That is, in part, also due to the wide availability of these medications as OTC drugs, the fact that they are available for free in the Brazilian public health system and that dispensation of opioids in Brazil are much more controlled than NSAIDs, even though both are prescription-regulated.^(9)^

In general, this study was successful in extracting data from a large number of patients using machine-learning technology (NLP) from a clinical database, showing accurate results and answering relevant questions from the drug use and population profiling perspectives, demonstrating the technology’s ability to extract relevant information from unstructured clinical notes. This infrastructure can increase gains as therapeutic options and access to health information.^(12)^ The authors showed the relevance that more automated means of leveraging unstructured data from daily clinical practice is crucial as therapeutic options and access to individual-level health information increase.^(12)^

Metamizole was by far the most common drug recorded in any age category. The distribution by age category was similar for each type of drug use. Fever remained the most common symptom in the pediatric population, whereas headache was the most frequent symptom in adults.

One limitation of this study was that the strength of applying NLP technology was limited by data availability and human support constraints. Further, the process of medical documentation could also be considered a limitation for the second cohort analysis, given that a considerable number of patients with symptoms of interest did not have a prescription in the EHR, with a paper prescription out of the system.

For this reason, we have to consider that the population without drug in a second cohort is probably not reliable (64% of the patients (n=12,650) without drug recorder) and should not mean a trend. This could be considered an important quality impact in the study, with relevant limitation involving dates and periods of each drug use, since these records are not precise. The results were limited to report on patients that had records for one single medication or for multiple drugs, but not determining the order of use or concurrent use.

In addition to this limitation, the NLP process did not find any record of the most serious adverse event (agranulocytosis). Other authors claim that this drug causes fewer adverse effects than ASA.^(13)^ Metamizole-based drugs are widely used in different areas of the world, such as South America, South Africa, the Middle East and some European countries.^(14-16)^ Several studies have been conducted to assess its safety. According to the International Study of Agranulocytosis and Aplastic Anemia (Boston Study), published in 1986, there is no association with aplastic anemia. As for agranulocytosis, the risk is 1.1 cases per million users.^(17,18)^ According to Ibañez et al. this risk increases with the duration of the treatment and disappears ten days after the last dose administered.^(19)^ At a meeting sponsored by the National Health Surveillance Agency (ANVISA - *Agência Nacional de Vigilância Sanitária*) on the safety of dipyrone, it was concluded that dipyrone, when compared to other analgesics/antipyretics on the market, has the necessary safety and efficacy to continue be marketed in Brazil as over-the-counter drug (MIP - *medicamento isento de prescrição*).^(20)^

A final study limitation identified is the lack of covariables that would allow a more comprehensive description of demographic and social characteristics of metamizole use.

## CONCLUSION

The present real world study was successful at identifying a large population with documented use of metamizole and allowed mapping the most important use indications and behaviors concerning its utilization as a single drug or in association with other medications.

Among patients who used metamizole, the treatment of some type of pain was the most common reason for its use, followed by fever, with utilization as monotherapy.

Although real world data to evaluate therapeutic options by Brazilian patients is unusual, it should have its potential contribution to health science fully explored.
